# *HD-AGP*s as Speciation Genes: Positive Selection on a Proline-Rich Domain in Non-Hybridizing Species of *Petunia*, *Solanum*, and *Nicotiana*

**DOI:** 10.3390/plants8070211

**Published:** 2019-07-08

**Authors:** Tara D. Callaway, Anu Singh-Cundy

**Affiliations:** Biology Department, Western Washington University, Bellingham, WA 98225, USA

**Keywords:** TTS proteins, interspecific reproductive barrier (IRB), unilateral incompatibility, incongruity, speciation, sympatric, pistil, pollen, Solanaceae

## Abstract

Transmitting tissue-specific proteins (TTS proteins) are abundant in the extracellular matrix of *Nicotiana* pistils, and vital for optimal pollen tube growth and seed set. We have identified orthologs from several species in the Solanaceae, including *Petunia* *axillaris axillaris* and *Petunia* *integrifolia*. We refer to TTS proteins and their orthologs as histidine domain-arabinogalactan proteins (HD-AGPs). HD-AGPs have distinctive domains, including a small histidine-rich region and a C-terminal PAC domain. Pairwise comparisons between HD-AGPs of 15 species belonging to *Petunia*, *Nicotiana*, and *Solanum* show that the his-domain and PAC domain are under purifying selection. In contrast, a proline-rich domain (HV2) is conserved among cross-hybridizing species, but variant in species-pairs that are reproductively isolated by post-pollination pre-fertilization reproductive barriers. In particular, variation in a tetrapeptide motif (XKPP) is systematically correlated with the presence of an interspecific reproductive barrier. Ka/Ks ratios are not informative at the infrageneric level, but the ratios reveal a clear signature of positive selection on two hypervariable domains (HV1 and HV2) when HD-AGPs from five solanaceous genera are compared. We propose that sequence divergence in the hypervariable domains of HD-AGPs reinforces sympatric speciation in incipient species that may have first diverged as a consequence of pollinator preferences or other ecological factors.

## 1. Introduction

**Figure 1:**
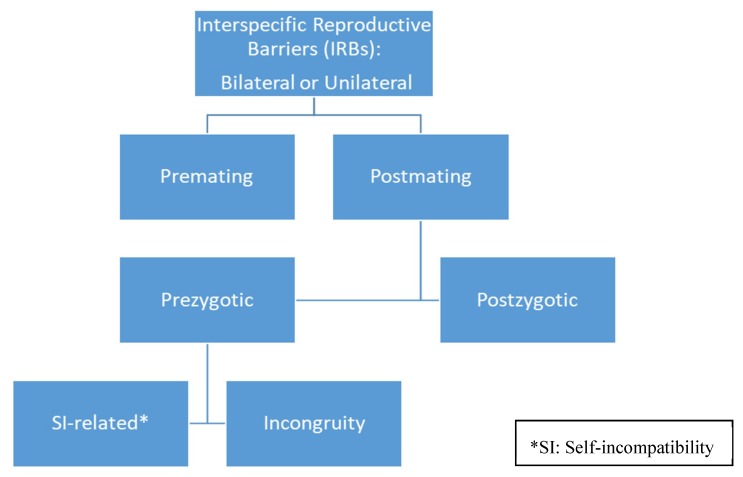
Interspecific reproductive barriers (IRBs) can operate at different levels in the path from pollen delivery to the formation of viable hybrid offspring. SI-related prezygotic IRBs are believed to rely on components of the self-incompatibility outbreeding system to actively inhibit heterospecific pollen on or in the pistil of the female partner. Incongruity encompasses all other prezygotic IRBs, and is thought to rely on passive mechanisms that manifest as suboptimal pollen performance in a heterospecific pistil. Incongruity includes conspecific pollen precedence, in which conspecific pollen tubes grow much better in the pistil than do heterospecific pollen tubes. At the mechanistic level, incongruity is the least understood of all the IRBs.

Interspecific reproductive barriers (IRBs) reduce the odds of successful hybridization between closely related taxa [1]. IRBs are bilateral when the two species fail to make successful hybrids, no matter which species functions as the female or male parent. Unilateral IRBs are also known: Hybridization occurs when the one species serves as the female (or male) parent, but the reciprocal cross fails to produce successful hybrids. The terminology associated with the different types of IRBs varies in the literature. Figure 1 illustrates IRBs as they are classified in this article.

The different types of IRBs are classified on the basis of the time at which they act in the reproductive process. At the broadest level, IRBs are of two main types: Premating or postmating. Geographical isolation, flowering phenology, ecological factors such as soil type, or ethological factors such as pollinator preference, can generate premating IRBs. Post-mating IRBs are of two types: Prezygotic and post-zygotic. Prezygotic IRBs involve one or more of the many steps in pollen-pistil interactions that begin with receipt of pollen on the stigmatic surface of the pistil and culminate with fertilization in the ovary. Postzygotic IRBs act after fertilization has occurred and could be caused by zygote inviability, embryo or endosperm failure, or the failure of the hybrid seedling to thrive.

IRBs play a key role in the evolution of species because they strengthen species boundaries by reducing or blocking hybridization between incipient species. However, not all IRBs succeed in fully isolating sister species. Incomplete isolation can generate interspecific hybrids with reduced fitness if the two parental species are highly adapted to different ecological niches or display distinctly different pollination syndromes [2]. To prevent the loss of reproductive potential to maladaptive hybrids [3], natural selection favors reduced gene flow between emerging lineages through a secondary isolation process, known as reinforcement [4,5]. Speciation reinforcement refers to natural selection against hybrid formation between diverging lineages that are incompletely separated by existing IRBs [2].

Identifying genes involved in generating IRBs is critical for understanding speciation. A speciation gene is defined as ‘any gene contributing to the evolution of reproductive isolation’ [6]. Speciation genes are predicted to show little within-population variation, but to evolve rapidly during speciation events, generating signs of positive (adaptive) selection in across-taxa comparisons. Genes involved in initiating speciation, and those that reinforce speciation, can both qualify as speciation genes [7].

Pollinator-driven premating IRBs are common in the Solanaceae, and genes controlling floral traits such as petal color, corolla shape, nectar production and release of volatile scent molecules can function as speciation genes [8]. For example, Hoballah et al. [9] identified a transcription factor that controls flower color in the petals of *Petunia* species, and they demonstrated that sequence variation in the *AN2* gene generates divergent pollination syndromes that alter pollinator preference and thereby isolate populations.

Complete pollinator fidelity is uncommon, therefore, speciation driven by pollinators generally does not produce absolute reproductive isolation [10]. Dell’Olivo et al. [11] studied sympatric populations of *Petunia axillaris* and *Petunia integrifolia* in Uruguay, and found that pollinator preference generates a strong premating IRB, but not an absolute one. They measured the strength of all potential IRBs between the two species, and reported that post-mating pre-fertilization IRBs were almost as strong as the pollinator-mediated isolation. Post-mating IRBs assume importance in sympatric species in particular because they can reinforce incipient speciation generated by pollinator preference, or other premating IRBs, to block gene flow completely.

Post-mating pre-fertilization IRBs involve pollen-pistil interactions. Self-incompatibility (SI) is the best-studied example of pollen-pistil interactions. SI is an outbreeding mechanism that prevents genetically related pollen from effecting fertilization. Gametophytic SI in the Solanceae is controlled by the *S*-locus, which includes a pistil-expressed S-RNase gene and pollen-expressed F-box genes [12].

Pistil proteins other than S-RNases are known to be necessary for SI. An arabinogalactan protein known as the 120 kDa protein is thought to mediate trafficking of S-RNases within the pollen tube [13]. An asparagine-rich protein (HT-A or HT-B) is also taken up by pollen tubes from the pistil extracellular matrix, and it associates with microsomes to help mediate SI through an unknown mechanism [14]. A stigma-specific protease inhibitor (NaStEP) that is internalized by both compatible and incompatible pollen tubes has also been shown to be necessary for SI [15].

That SI has a role in interspecific reproductive barriers was suspected from the SI X SC rule, reported for diverse taxa displaying either gametophytic or sporophytic SI [16]. According to the SI X SC rule, when a self-incompatible (SI) female partner is crossed with a heterospecific self-compatible (SC) male, the SI pistil rejects pollen from the SC species, but the reciprocal heterospecific cross is successful in setting seed. This phenomenon is termed unilateral incompatibility (UI) in much of the literature on the subject, but we have avoided the term because of the potential for confusion between conspecific and heterospecific pollen-pistil interactions and because the term ‘hybrid incompatibility’ is commonly used for some types of post-zygotic IRBs.

The SI X SC rule is illustrated by some SI and SC *Nicotiana* species. Cruz-Garcia et al. [17] showed that S-RNases are causally involved in the rejection of pollen from self-compatible *Nicotiana* species on pistils of self-incompatible *Nicotiana alata*. Genetic knockdown of HT-A/HT-B also causes loss of SI together with an inability to reject heterospecific pollen unilaterally [18].

There are many exceptions to the SI X SC rule. Instead of always operating unidirectionally, SI-related IRBs can operate bilaterally between two SI species. Furthermore, prezygotic IRBs are known to exist between self-compatible accessions of otherwise SI species or between sister taxa not known to possess functional SI [16]. Baek et al. [19] report that conspecific pollen precedence, rather than an SI-related IRB, blocks the hybridization between two SI species of *Solanum*: *Solanum habrochaites* and *Solanum arcanum*.

In contrast to SI-related IRBs, genes underlying incongruity are poorly understood. They are likely to include genes that support pollen germination, normal pollen tube growth, and targeting of ovules by pollen tubes growing in compatible pistils [17]. At the molecular level, *PELPIII* is one of the few genes that has been implicated in incongruity. The encoded protein, PELPIII, is structurally similar to the 120 kDa protein and is taken up by pollen tubes and incorporated into the cell wall [17]. Noyszewski et al. [20] found that knocking down PELPIII expression in *Nicotiana tabacum* pistils enabled successful pollination by *Nicotiana repanda* and *Nicotiana obtusifolia*, which are otherwise isolated from *N. tabacum* by IRBs.

Because they are known to influence pollen tube growth, transmitting tissue-specific proteins (TTS proteins) should also be evaluated as candidate speciation genes. These arabinogalactan proteins were the first proteins demonstrated to play a direct role in promoting pollen tube growth in the style of *Nicotiana tabacum* and *N. alata* [21]. Pollen tube growth was reduced, and seed set was low in transgenic plants in which TTS gene expression had been knocked down using antisense RNA.

Orthologs of TTS proteins were identified in *Petunia hybrida* by Twomey et al. [22], who also demonstrated that the TTS proteins of *N. tabacum* are expressed in seedlings, roots, and leaves of tobacco. The *P. hybrida* ortholog (*PhPRP1*) is likewise expressed in vegetative tissues, as well as in the transmitting tissue, which constitutes the pollen tube pathway in the central core of a solanaceous style. Because TTS protein and its orthologous proteins are not unique to the transmitting tissue, Twomey et al. [22] refer to TTS proteins and their orthologs as histidine-domain arabinogalactan proteins (HD-AGPs). HD-AGPs contain a histidine-rich domain not seen in any other family of arabinogalactan proteins (AGPs) described so far. A proline-rich domain characterized by tetrapeptide repeat motifs (XKPP, where X can be A/V/T) is another distinctive feature of HD-AGPs. The C-terminal half of the protein contains a PAC domain (also known as the Ole-1 domain) found in several other types of AGPs, including the 120 kDa protein and PELPIII [23].

The goal of this study was to investigate *HD-AGP*s as candidate speciation genes. We hypothesized that sequence divergence in *HD-AGP*s generates post-mating prezygotic IRBs that would reinforce sympatric speciation in incipient sister lineages. The main predictions of the hypothesis were that (1) the signature of positive selection would be detected on any protein domain potentially involved in species-specific pollen-pistil interactions, and (2) sequence divergence in this domain would correlate with incongruity between sister taxa.

We compared *HD-AGP* domains of species-pairs within *Petunia*, *Solanum*, and *Nicotiana*. In comparing 5 species and sub-species of *Petunia*, we found that HD-AGPs are identical or near-identical in species that cross-hybridize readily, but XKPP indels in the HV2 domain are associated with interspecific incongruity. In these *Petunia* species, interspecific incongruity manifests as an inability to enter the rapid phase of pistil-dependent pollen tube growth, resulting in failure to set seed. Variation in XKPP motifs also predicts pollen-pistil incongruity in *Nicotiana* and *Solanum*. The ratio of the rate of non-synonymous/synonymous substitutions (Ka/Ks) reveals positive selection on the HV1 and HV2 domains and purifying selection on all other domains in intergeneric comparisons. We propose a model for incongruity-mediated reinforcement of speciation in lineages first isolated in sympatry by ecological factors such as differences in flowering phenology or pollinator preference.

## 2. Results

### 2.1. Breeding Behavior in Petunia Accessions

**Figure 2:**
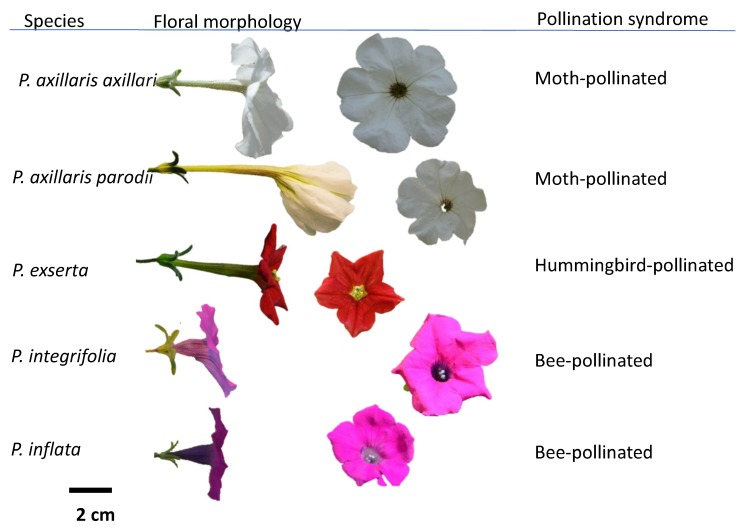
Flower size and morphology in five species and subspecies of *Petunia*. The pollination syndrome characteristic of each taxon is shown in the column on the right. The scale bar equals two centimeters.

Our accessions of *Petunia axillaris axillaris* and *Petunia axillaris parodii* are self-compatible. Our accessions of *P. integrifolia* and *P. inflata* exhibit strong self-incompatibility. Members of the *P. axillaris* complex (*Petunia axillaris axillaris* and *Petunia axillaris parodii*) are reproductively isolated from *P. integrifolia* as well from *P. inflata* because cross-pollination fails to set seed in either direction. These patterns of breeding behavior are concordant with prior studies of natural populations [24]. Floral morphology of these 5 species/subspecies of *Petunia* is shown in Figure 2.

**Figure 3:**
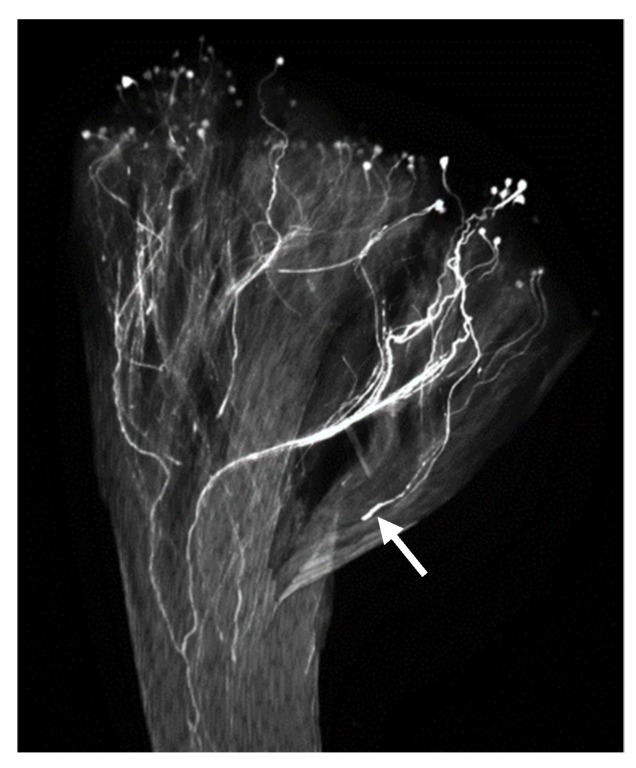
Morphology of *P. axillaris axillaris* pollen tubes growing in *P. integrifolia* pistils for 24 h. The arrow points to the dilated tips with heavy sub-terminal deposits of callose.

To understand the nature of the bilateral IRBs between the *Petunia* species, we used fluorescence microscopy to examine pistils pollinated for 24 h with conspecific or heterospecific pollen. We found that *Petunia axillaris axillaris* pollen tubes are inhibited in the upper one-third of *P. integrifolia* pistils and they exhibit morphologies typical of rejected pollen tubes, due to self-incompatibility, including dilated tips with heavy sub-terminal callose deposits (Figure 3). In contrast, *P. axillaris axillaris* is completely interfertile with *P. axillaris parodii* and also with *P. exserta* [24], and in all cases, pollen tubes reach the base of the style by 30 h.

### 2.2. Time Course of Conspecific and Interspecific Pollination

**Figure 4:**
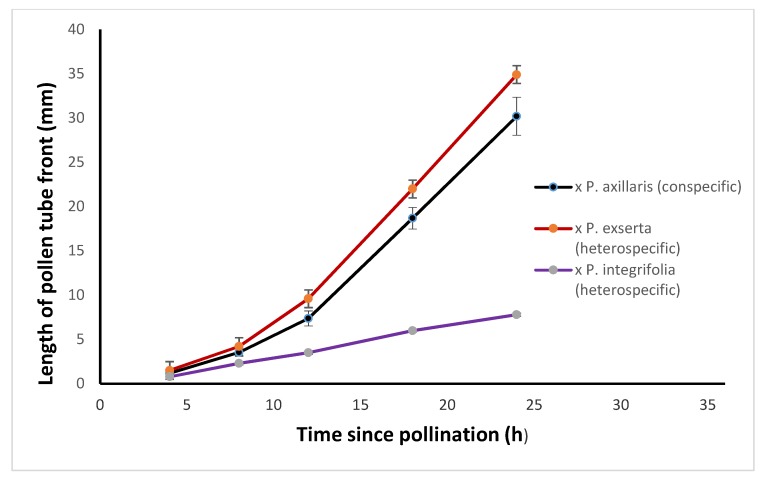
Time course of pollen tube growth in *P. axillaris axillaris* pistils following cross-pollination with conspecific pollen, and two types of heterospecific pollen (from *P. exserta* or *P. integrifolia*). Error bars show standard deviation of measurements from six pollinated pistils. The pollen tube front is defined as the length of the longest bundle of pollen tubes that contains 20 or more pollen tubes.

There is biphasic growth of conspecific pollen tubes in the pistils of *Petunia*, with a pronounced acceleration of the growth rate by 12 h post-pollination [25]. Biphasic pollen tube growth is seen in *P. axillaris* pistils after conspecific pollination, but *P. exserta* pollen also performs well in *P. axillaris* pistils and their pollen tubes display the same pattern of biphasic growth as conspecific pollen tubes (Figure 4).

As seen in Figure 4, the transition to rapid growth fails to occur when *P. axillaris* pistils are pollinated with *P. integrifolia* pollen. The heterospefic pollen continues to grow slowly for at least 72 h, by which time pollinated flowers start to exhibit floral senescence. Seed set fails in this interspecific pollination presumably because the pollen tube growth transition [25] fails to occur. The dynamics of heterospecific pollen tube growth are consistent with incongruity between *P. axillaris* pistils and *P. integrifolia* pollen, and it appears to be the underlying cause of the reproductive barrier between the two species when the former is the pistillate partner. Thus, different reproductive barriers operate depending on which species is the pistillate parent: SI-related IRB when *P. integrifolia* is the female partner, incongruity when *P. axillaris* is the female partner.

### 2.3. HD-AGPs Sequences from Petunia Species

*HD-AGP* genes have been described from *P. axillaris parodii* and from *P. inflata* by Brooks [26], and genomic sequences are also available at the Solgenomics database [27]. To identify the latter, we conducted a BLAST search at the Solgenomics site, using the *HD-AGP* from *P. axillaris parodii* (*Pap*PRP1) and from *P. inflata* (*Pi*PRP1). The sequence search enabled us to identify a scaffold containing an *HD-AGP* from *P. axillaris* (Peaxi162Scf00250g00029), and one containing the *HD-AGP* genomic sequence from *P. inflata* (Peinf101Scf00169g23008). Both orthologs are present as a single copy in their respective genomes, and each has a single intron located at the same position, but differing substantially in size (Table 1).

We used RT-PCR to isolate and clone an HD-AGP ortholog, *Paa*PRP1, from *P. axillaris axillaris.* Using the same methods, we cloned another ortholog, *Pit*PRP1, from *P. integrifolia*. *PaaPRP1* is a 768 bp complete cDNA clone, and *PitPRP1* is a 780 bp complete cDNA clone. Both mRNA sequences have a 27 bp 5′-untranslated region that is conserved. Sequence alignments show that the polypeptide encoded by *PaaPRP1* consists of 256 amino acids, and *PitPRP1* encodes a polypeptide that is 260-amino acid in length.

Brooks [26] described an HD-AGP ortholog (*Pe*PRP1) from the only hummingbird-pollinated petunia, *Petunia exserta*. In order to obtain the intron sequences, we used gene-specific primers to PCR-amplify genomic DNA. Sequencing of the amplicons revealed the presence of a single 593 bp intron in *Pe*PRP1 and a single 604 bp intron in *PitPRP1* (Table 1). Likewise, we amplified and sequenced introns from genomic DNA corresponding to *PaaPRP1* and *PiPRP1*. Comparison of the intron sequences enabled us to infer that the ‘*P. axillaris*’ genome deposited at the Solgenomics site comes from *P. axillaris axillaris*, although the subspecies is not identified at that genome repository.

### 2.4. Domain Structure of Petunia HD-AGPs

**Figure 5:**
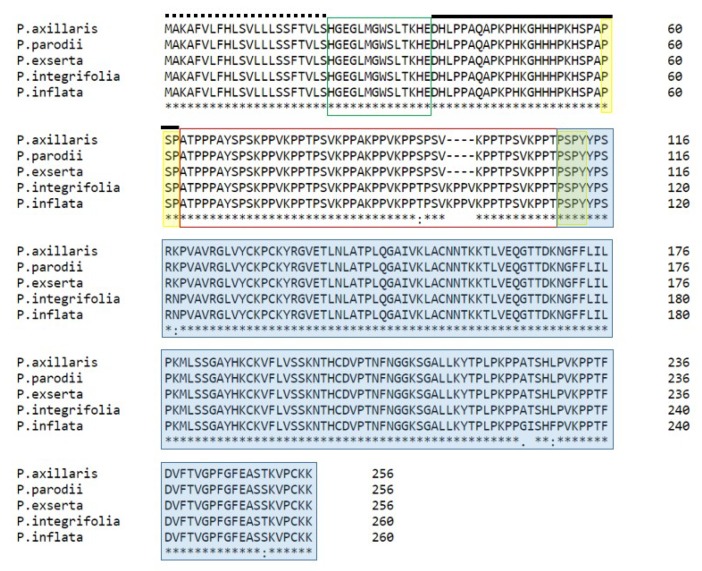
Multiple alignments of HD-AGP protein sequences from *P. axillaris axillaris* (*Paa*PRP1), *P. axillaris parodii* (*Pap*PRP1), *P. exserta* (*Pe*PRP1), *P. integrifolia* (*Pit*PRP1), and *P. inflata* (*Pi*PRP1). The numbers at the end of the row indicate amino acid positions. The signal peptide sequence is shown with a dotted line above it; the two hypervariable domains, HVI and HV2, are boxed in green and red, respectively; a black line lies over the histidine-rich domain; the highly conserved PAC/Ole-1 domain is highlighted in blue. The residues highlighted in yellow are conserved in all solanaceous HD-AGPs described so far, and they flank the HV2 domain in all HD-AGPs.

All five *Petunia* HD-AGPs display the domain architecture characteristic of HD-AGPs [22]. The domains include: A highly conserved signal peptide sequence; HV1, a short N-terminus domain that tends to vary when sequences from phylogenetically diverse taxa are compared; a histidine-rich domain that is 29 residues in length and highly conserved among *Petunia* HD-AGPs; HV2, a proline-rich domain, with multiple XKPP tetrapeptide motifs; and a C-terminal PAC/Ole-1 domain (Figure 5). Nucleotide sequence alignments are available in Supplementary Materials, Figure S1.

*PiPRP1* and *PaaPRP1* display 97.7% nucleotide sequence similarity, but there is a total of 18 nucleotide substitutions between them (Figure S1). The variant residues include a 12-nucleotide indel (insertion/deletion) in *PiPRP1* that is not seen in *PaaPRP1*. The 12-nucleotide indel codes for ‘VKPP’ in the HV2 domain of *Pi*PRP1 (Figure 5). XKPP motifs, in which ‘X’ represents A/V/S/T residues, are abundant in the proline-rich HV2 domain and are one of the distinctive features of HD-AGPs, setting these proteins apart from other pistil-expressed AGPs. This specific ‘VKPP’ motif occurs in the HD-AGPs of both *P. integrifolia* and *P. inflata*, but is absent in the HD-AGPs of the *P. axillaris* complex and *P. exserta*. Because the former is believed to be the ancestral clade, we infer that the tetrapeptide was deleted in *PaaPRP1* as the *P. axillaris* complex diverged from the ancestral group.

### 2.5. Tetrapeptide Indels Are Associated with Reproductive Barriers

XKPP indels make up a majority of the sequence variation exhibited by HD-AGPs of incongruous species-pairs (Table 2). We found no XKPP indels in comparing HD-AGPs from species that are not isolated by reproductive barriers. In contrast, XKPP indels account for 50% or more of all the variant residues in the entire protein when HD-AGPs from non-hybridizing species are compared.

### 2.6. Ka/Ks Metric Lacks Sensitivity to Detect Evolutionary Changes in Closely-Related Taxa

Evolutionary change in proteins is often evaluated by comparing the rate of non-synonymous change per non-synonymous site (Ka) to the rate of synonymous change per synonymous site (Ks). A Ka/Ks ratio below one is indicative of negative or purifying selection to maintain protein function. A Ka/Ks ratio of 1 represents neutral selection, and a ratio above one signifies positive selection resulting from sequence divergence that has adaptive value.

We found that using Ka/Ks ratios to evaluate selection is non-informative in closely related taxa such as the *Petunia* species. Because there is relatively little sequence variation among these sequences as a whole, Ks is sometimes zero just by chance, which makes the ratio an undefined quantity. We represent this outcome as Ka-Ks > 0. When sequences from species isolated by reproductive barriers are compared, the Ka-Ks > 0 result may be taken as a tentative indicator of positive selection on the HV2 domain. However, the presence of XKPP indels is an even stronger signal for Darwinian selection on the HV2 domain because the indel coincides 100% with the presence of reproductive barriers between the species-pair being compared (Table 3).

### 2.7. Comparison of HD-AGPs from Nicotiana

To investigate whether the correlation between HV2 sequence divergence and IRBs holds for other taxa in the Solanaceae, we examined all *Nicotiana* species for which we could find sufficient information. The latter comprised two criteria: Availability of breeding behavior data, and the availability of full-length HD-AGP sequence. We retrieved HD-AGP sequences for five *Nicotiana* species from the Solgenomics repository and from contributions by Noyszewski et al. [20] at NCBI. Each of the five belongs to a different section of the genus, and some even have different ploidy levels (Table 4). Though they belong to different sections of the genus, *N. tomentosiformis* and *N. obtusifolia* can hybridize with each other in both directions. *N. sylvestris* and *N. paniculata* also belong to different sections of the genus, but can accept pollen from each other and from *N. alata*. These species-pairs, therefore, constituted an even more stringent test of our hypothesis.

**Figure 6:**
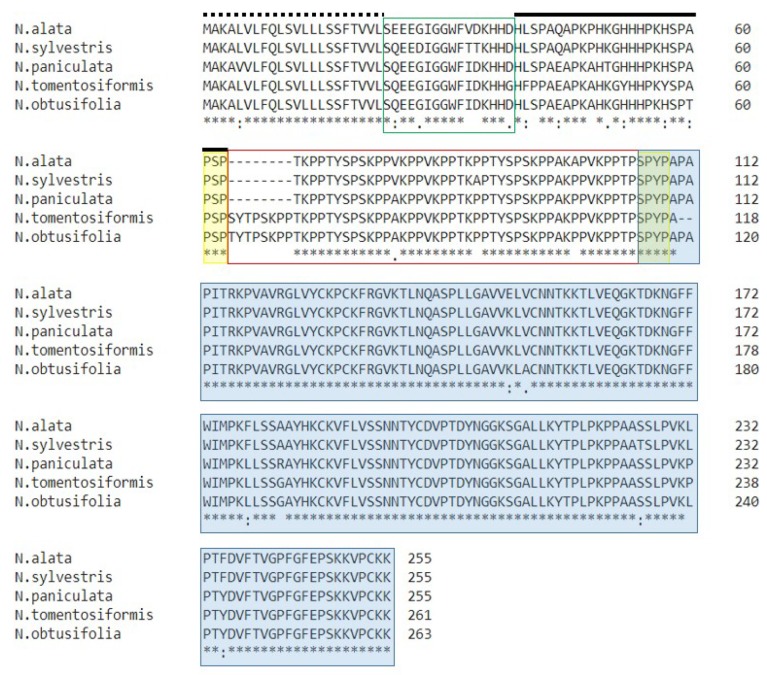
Multiple alignments of HD-AGP protein sequences from *Nicotiana alata* (*Na*PRP4), *N. sylvestris* (*Ns*PRP1), *N. paniculata* (*Np*PRP1), *N. tomentosiformis* (*Nto*PRP1) and *N. obtusifolia* (*Nob*PRP1). The signal peptide sequence is shown with a dotted line above; the two hypervariable domains, HVI and HV2, are boxed in red; a black line lies over the histidine-rich domain; the highly conserved PAC/Ole-1 domain is highlighted in blue.

*N. alata* is typically self-incompatible and is known to reject heterospecific pollen through SI-related IRB (also known as unilateral incompatibility or UI), with a requirement for functional S-RNase and HT-A genes [13,18]. *Nicotiana paniculata* produces seed when pollinated with *N. alata* pollen, according to Christoff [29]. Although the overall sequence similarity between HD-AGPs from *N. alata* and *N. paniculata* is only 95%, their HV2 sequence is remarkably similar, with no XKPP indels (Figure 6). However, a single nucleotide substitution appears to have converted one of the AKPP motifs to an AKAP motif in *Na*PRP4 from *N. alata* (Supplementary Materials, Figure S1). The functionality of the HV2 domain is likely not altered by the small substitution, which would account for the lack of incongruity when this species serves as the pistillate parent for *N. alata* pollen.

*N. sylvestris* does not set seed when pollinated with *N. alata* pollen [29]. The overall sequence similarity between HD-AGPs from *N. alata* and *N. sylvestris* is 96%. Although there are no XKPP indels in their HV2 domains, two XKPP motifs are divergent between them. A TKPP motif in *Na*PRP4 is converted into a TKAP motif in *Ns*PRP1 from *N. sylvestris*, and an AKPP motif in *Ns*PRP1 has mutated into AKAP in *Na*PRP4. Non-synonymous mutations in two XKPP motifs and the exceptionally long pistils of *N. sylvestris* [30] may together constitute an effective barrier to fertilization by *N. alata* pollen.

*N. obtusifolia* (syn. *N. trigonophylla*) does not set seed with *N. alata*, *N. paniculata*, or *N. sylvestris* pollen [29]. Consistent with our working hypothesis, the *N. obtusifolia* HD-AGP sequence is markedly different in the HV2 region in that it contains an 8-residue indel which includes an XKPP motif (Figure 6). The HD-AGPs of *N. obtusifolia* and *N. tomentosiforis* are only 95% identical overall (Table 5), but their HV2 domains are virtually identical (Figure 6). As predicted by the hypothesis, interspecific crosses between the two are successful and produce viable hybrid seedlings [31].

### 2.8. Comparison of HD-AGPs from Solanum

**Figure 7:**
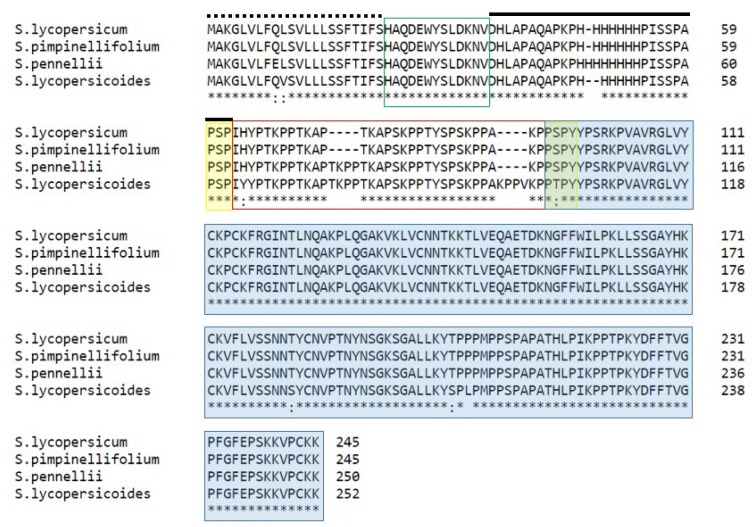
Multiple alignments of HD-AGP protein sequences from *Solanum lycopersicum* (*Sl*PRP1), *S. pimpinellifolium* (*Spp*PRP1), *S. pennellii* (*Sp*PRP1), and *S. lycopersicoides* (*Sly*PRP1). The signal peptide sequence is shown with a dotted line above; the two hypervariable domains, HVI and HV2, are boxed in green and red, respectively; a black line lies over the histidine-rich domain; the highly conserved PAC/*ole-1* domain is highlighted in blue.

To investigate whether the hypothesis is supported in the largest genus within the Solanaceae, we investigated all *Solanum* taxa for which we could find sufficient information, namely, breeding behavior data and full-length HD-AGP sequences. We retrieved HD-AGP orthologs from *Solanum lycopersicum* (XP_004232888.1) and *S. pennellii* (XM015208828) from the NCBI database of non-redundant genes, and *S. pimpinellifolium* (Sopim02g078100) and *S. lycopersicoides* (SLYD0.6ch02:68819601-68821800) from the Solgenomics repository. Nucleotide sequence alignments are available in Supplementary Materials, Figure S1. Multiple alignments of the four *Solanum* HD-AGPs polypeptides (Figure 7) reveals that they share the same domain architecture as the *Petunia* and *Nicotiana* HD-AGPs (Figure 5 and Figure 6).

The two red-fruited species of tomato, *S. lycopersicum* and *S. pimpinellifolium*, cross-hybridize easily in both directions with high seed set [32]. As seen in Figure 7, their HD-AGP sequences are 100% identical, with no divergence in the number and location of the XKPP motifs.

*S. pennelli* is a green-fruited self-compatible wild tomato that displays IRB in crosses with the red-fruited species: It “rejects” *S. lycopersicum* pollen in an SI-independent manner, but the reciprocal cross yields seed in artificial pollinations [33]. We compared HD-AGP sequences for *S. lycopersicum* and *S. pennellii*, and as shown in Figure 7, their amino acid sequences are similar except for a TKPP indel in the HV2 domain. *S. lycopersicoides* is reproductively isolated from all three of the other *Solanum* and its HD-AGP displays XKPP motif variants when compared with HD-AGPs from the other three (Table 6).

### 2.9. Comparison of HD-AGPs from Petunia, Nicotiana, Solanum, and Capsicum

**Figure 8:**
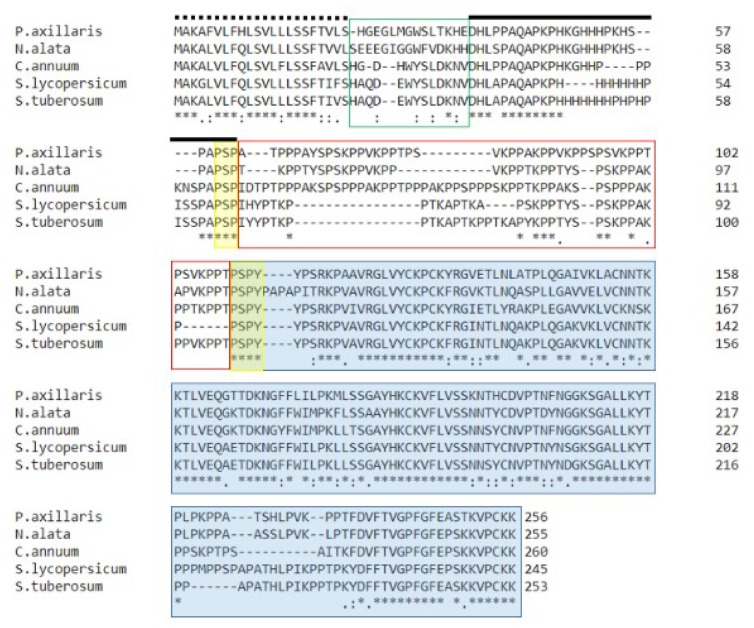
Contractions and expansions of XKPP motifs are the most striking feature of divergence in HD-AGP sequences from five divergent genera of the Solanaceae. The figure shows multiple alignments of HD-AGP protein sequences with manual adjustments of gaps in MEGA. The sequences are from five divergent species within the Solanaceae: *P. axillaris* (*Paa*PRP1), *Nicotiana alata* (*Na*PRP4), *Capsicum annuum* (*Ca*PRP1), *Solanum lycopersicum* (*Sl*PRP1), and *Solanum tuberosum* (*Stu*PRP1). The signal peptide sequence is shown with a dotted line above; the two hypervariable domains, HVI and HV2, are boxed in red; a black line lies over the histidine-rich domain; the highly conserved PAC/Ole-1 domain is highlighted in blue.

How do solanaceous HD-AGPs compare across diverse genera within the family? Is positive selection detectable by the most widely used metric, the Ka/Ks ratio, among these presumably disparate HD-AGPs? To answer these questions, we found two additional full-length HD-AGP sequences from public databases: One from hot peppers (*Capsicum annuum*) and one from potato (*Solanum tuberosum*). We compared the HD-AGPs from these well-studied species (each with a genome project of its own) to an HD-AGP representative from the best-studied member of the other genera (*Petunia*, *Nicotiana*, and *Solanum*). We created a multiple alignment of HD-AGPs from these 5 divergent taxa, including an HD-AGP from *Capsicum annuum* (AY533017.1) and from *Solanum tuberosum* (XM_006346963). As seen in Figure 8, the signal peptide and His domain display relatively little variability even among these phylogenetically diverse taxa that have been separated for at least 30 million years [34]. The PAC domain is largely conserved except for a short stretch near the C-terminus. In contrast, the HV2 domain displays significant sequence divergence that is strongly biased toward contractions or expansions of XKPP motifs.

Analysis of mutation rate via Ka/Ks ratios was far more informative for these phylogenetically diverse taxa than it was for sister species within a genus (see Table 3). Ka/Ks ratios were calculated for pairwise comparisons of the five HD-AGP orthologs using the DnaSP program. The Ka/Ks ratios confirm purifying selection in the signal peptide, histidine-rich domain and PAC domains (Table 7).

In nine pairwise comparisons, eight showed positive selection in either HV1 or HV2 or in both, as indicated by a Ka/Ks ratio in excess of one. The exception was the *Capsicum annuum* HD-AGP compared to the tomato HD-AGP, which had a Ka/Ks = 0.77 for the HV2 region. However, the HV2 domains of these two proteins are the most divergent among all the pairwise comparisons in this table, with 18 variant residues, including two XKPP indels (Figure 8).

### 2.10. Association of XKPP Variants and IRBs in 15 Species-Pairs

We conducted nonparametric two-tailed tests of association to test the hypothesis that XKPP variants are better predictors of reproductive barriers than is the overall amino acid similarity between the HD-AGPs of a pair of solanaceous species. We created a matrix of species-pairs, and recorded the absence (0) or presence (1) of reproductive barriers between them, which constituted the dependent variable (Supplementary Materials, Table S1). One independent variable we tested was the absence (0) or presence (1) of any XKPP variants (caused by indel or substitution). The other independent variable we investigated was the percent amino acid identity between the HD-AGPs of the species pair being compared.

In assessing the link between XKPP variants and the presence of reproductive barriers, Spearman’s Rho returned a coefficient of 0.915, significant at the 0.0001 level. In contrast, percent amino acid identity was less strongly associated with the presence of reproductive barriers, with a coefficient of −0.394, significant at 0.051. The negative direction of the association was expected because reproductive barriers are expected to grow as percent similarity decreases. The striking aspect of this analysis is that XKPP variants predict reproductive barriers with a higher level of confidence than does the overall amino acid similarity between the HD-AGPs of the species being compared.

### 2.11. HD-AGPs Exhibit Low within-Species Polymorphism

Speciation genes are predicted to show very little within-species polymorphism, because within panmictic populations they are expected to be under purifying selection to maintain gene function. To assess whether HD-AGPs display this predicted hallmark of speciation genes, we compared infraspecific polymorphism in HD-AGPs from multiple *P. axillaris axillaris* populations and also from multiple *P. inflata* populations. We compared HD-AGP sequences from two different accessions of *P. axillaris axillaris* and also from the *P. axillaris* genomic sequence returned by our BLAST search at Solgenomics (Peaxi162Scf00250). The three independent sequences are 99.8% identical at the nucleotide level, suggesting very low polymorphism at the infraspecific level. Similarly, the *P. inflata* HD-AGP sequenced by Brooks [26] is 99.4% identical to the sequence returned by BLAST search of the *P. inflata* genome at Solgenomics (Peinf101Scf00169g23008.1).

Sequences encoding TTS-2 in *Nicotiana tabacum* were first reported by Cheung et al. [28], and subsequently, sequences encoding these proteins have been submitted to GenBank by Coates et al. (accession number EB427955) and by Goldman et al. (accession number FG629528). A comparison of the error-free portions of the publicly available sequences reveals 99.8% nucleotide sequence identity among the TTS-2 sequences. Thus, *Nicotiana* HD-AGPs parallel the low polymorphism we observed in the two *Petunia* species.

### 2.12. The HD-AGP Gene Tree Is Broadly Consistent With Well-Known Phylogenies

**Figure 9:**
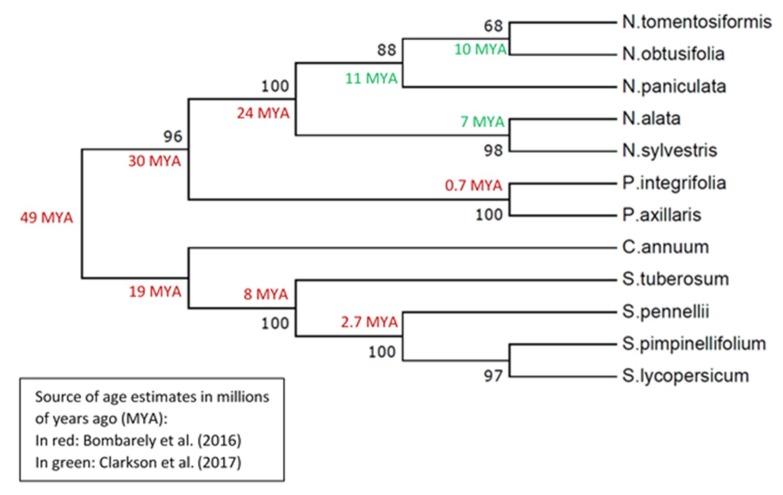
Phylogenetic reconstruction of HD-AGP proteins using the UPGMA statistical method in the MEGA7 package with 1000 bootstrap replications. The genetic distances were computed using the Tamura-Nei model and are in the units of the number of base substitutions per site. The divergence time estimates, where available, are from two published sources: From Clarkson et al. [36] for the *Nicotiana* species and from Bombarely et al. [27] for some of the remaining species. The age of some of the crown species is difficult to estimate because they are very young lineages.

We used neighbor-joining, maximum likelihood, and UPGMA methods to infer gene trees for HD-AGPs from *Petunia axillaris axillaris*, *P. integrifolia*, five *Nicotiana* species, 4 *Solanum* species and *Capsicum annuum*. The overall topology of the resulting gene trees matches the well-known phylogenies reported for these solanaceous genera (Figure 9), except that most species trees place *Nicotiana* as sister to the *Solanum* clade [35] rather than to the *Petunia* clade.

**Figure 10:**
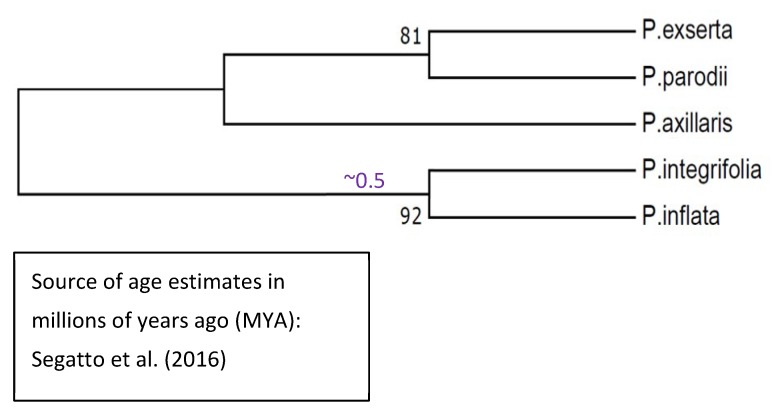
Phylogenetic reconstruction of HD-AGPs from five *Petunia* taxa. We used the UPGMA statistical method in the MEGA7 package with 1000 bootstrap replications. The genetic distances were computed using the Tamura-Nei model and are in the units of the number of base substitutions per site. The divergence time estimates, where available, are derived from Segatto et al. [37].

We did not include all species and subspecies of *Petunia* in the overall gene tree shown in Figure 9 because doing so generates an unbalanced tree, given that subspecies are included among these *Petunia* taxa but not among the other solanaceous taxa. Instead, we deduced the gene tree for *Petunia* HD-AGPs separately, as shown in Figure 10.

The gene tree of *Petunia* HD-AGPs is concordant with the published phylogeny of these taxa [38], except that the clustering of *P. exserta* and *P. axillaris parodii* (Figure 10) may be an artefact of the nearly identical HD-AGP sequences between the *P. axillaris* complex and *P. exserta*. Segatto et al. [37] have noted that *P. axillaris axillaris* is broadly distributed in South America, and its range overlaps with that of *P. exserta*, which is an ecologically threatened endemic found only in a few rock shelters in southern Brazil. Their ecogeographical proximity would suggest that *P. exserta* is more closely related to *P. axillaris axillaris* than to *P. axillaris parodii*, which is allopatric to *P. axillaris axillaris* and especially distant geographically from *P. exserta* [39]. Most published phylogenies show *P. exserta* in a polytomy with, or as sister to, the *P. axillaris* complex [40] instead of clustering with one or the other member of the complex. Thus, HD-AGP sequences are likely to be of little help in resolving branch tips with a very short evolutionary history and when no IRBs isolate the lineages in question.

## 3. Discussion

### 3.1. The Hypothesis: Sequence Divergence in HD-AGPs Generates Incongruity

Histidine-domain arabinogalactan proteins (HD-AGPs), such as TTS proteins from *Nicotiana* [28] and *Ph*PRP1 from *Petunia hybrida* [22], are abundant in the extracellular matrix of solanaecous pistils, and the proteins have been shown to promote pollen tube growth [41]. The goal of this study was to investigate whether sequence divergence in certain variable domains is linked to reproductive barriers in *Petunia*, *Nicotiana,* and *Solanum* species.

We hypothesized that all domains of HD-AGPs are under negative (purifying) selection within interbreeding populations, but speciation reinforcement selects for sequence divergence in a proline-rich hypervariable domain (HV2) that is likely to be involved in species-specific pollen-pistil interactions. The hypothesis predicts that species isolated by incongruity in pollen-pistil interactions will exhibit sequence variation in the HV2 domain of their respective HD-AGPs.

We cloned and sequenced HD-AGPs from *P. axillaris axillaris* and *P. integrifolia*, and compared these sequences with HD-AGP sequences from *P. axillaris parodi*, *P. inflata*, and *P. exserta* described by Brooks [26]. We also conducted BLAST searches at the Solgenomics website, which houses the genome database for *Petunia axillaris* and *P. inflata* together with the genome sequences of a number of other model organisms in the Solanaceae such as *Solanum* and *Nicotiana* [42]. The search revealed that a single gene encodes HD-AGPs in the genome of *P. axillaris* and *P. inflata*. Likewise, a BLAST search turns up a single hit in the genome of tomato (Solyc02g078100.3), *Capsicum* (CA02g15000) and eggplant (Sme2.5_02187.1_g00005.1), but two different scaffolds are returned in searching the genome of *Nicotiana benthamiana*, a known allopolyploid [43]. Thus, HD-AGP appears to be a single-copy gene in diploid members of the Solanaceae.

### 3.2. HD-AGPs Are Identical or Near-Identical in Cross-Hybridizing Petunia Species

HD-AGP sequences from the two sub-species of the *Petunia axillaris* group, *P. axillaris axillaris* and *P. axillaris parodii*, are 98.8% identical at the nucleotide level and 99.8% identical at the protein level. The two taxa are closely related and hybridize easily, but the two species are not lacking in genetic differentiation as indicated by the heterogeneity in intron size and sequence.

Natarella and Sink [44] were able to distinguish the two subspecies on the basis of distinctive peroxidase enzyme patterns. Sequence variation in *Hf1*, a nuclear gene that encodes flavonoid-3′,5′-hydroxylase, enabled Chen et al. [45] to distinguish the two subspecies and map their phylogenetic relationship with 17 other taxa in the genus. A gene encoding a cryptochrome/photolyase is only 89.3% identical between the two subspecies [46]. We conclude, therefore, that the conservation of the HD-AGP sequences of *P. axillaris axillaris* and *P. axillaris parodii* reflects evolutionary constraint to maintain protein function, rather than insufficient evolutionary time to accumulate mutations. The natural range of the two subspecies lies on opposite sides of Rio Negro in Uruguay and isolation by ecogeographic factors [24] likely sufficed to prevent homogenization of the taxa in the absence of any reproductive barriers.

*Petunia axillaris axillaris* and *P. exserta* are distinctly different species with very different pollination syndromes [47]. They hybridize readily in both directions, and as predicted by the hypothesis, their HD-AGPs are 100% identical at the amino acid level (Figure 4).

### 3.3. Tetrapeptide Indels in Petunia HD-AGPs Co-Vary with Interspecific Incongruity

The white-flowered moth-pollinated *P. axillaris* complex and red-flowered bird-pollinated *P. exserta* are reproductively isolated from both *P. integrifolia* and *P. inflata*, which produce purple flowers pollinated by bees and other daytime pollinators and exhibit strong self-incompatibility. *P. axillaris axillaris* pollen is rejected on *P. integrifolia* pistils with symptoms typical of SI (Figure 3). However, post-mating IRBs between these species do not follow the SI X SC rule because the reciprocal cross is also unsuccessful.

In compatible, conspecific pollination, pollen tube growth in *Petunia* pistils is biphasic, with a relatively slow growth rate in the first 8 h after pollination, followed by a rapid growth phase that coincides with the entry of pollen tubes into the style [25,48]. As seen in Figure 4, *P. integrifolia* pollen tubes grow more slowly in *P. axillaris* pistils, and the transition to rapid growth in the style fails to occur in this type of pollination. The slow rate of growth means that floral senescence ensues before the heterospecific pollen tubes can reach the ovary. Pollination failure in this cross (*P. axillaris* X *P. integrifolia*, with the former as a pistillate partner), therefore, qualifies as a case of interspecific incongruity.

Strikingly, the HD-AGP of *P. integrifolia* differs from the HD-AGP of *P. axillaris axillaris* in sequence, and the difference lies mainly in the proline-rich HV2 region and involves the deletion of an XKPP motif in *PaaPRP1* from *P. axillaris axillaris*. The proline-rich HV2 region is highly glycosylated, and the XKPP repeat motifs may serve as a recognition signal for the addition of a hydroxyl group on one of the prolines in the motif by prolyl hydroxylases localized in the endoplasmic reticulum. The subsequent transfer of arabinogalactan chains of variable length to the hydroxyproline, catalyzed by pistil-specific O-glycosylases, likely generates the well-known heterogeneity in molecular mass that pistil HD-AGPs display [22,28].

### 3.4. Incongruity in Nicotiana Too Is Linked with Variation in XKPP Repeat Motifs

The correlation between XKPP indels and reproductive barriers is even more striking in the *Nicotiana* group. That the *P. axillaris* complex and *P. exserta* interbreed readily might be ascribed to their phylogenetic closeness, which could also explain their nearly identical HD-AGP sequences. However, in the *Nicotiana* group, we find support for the hypothesis in crosses involving phylogenetically diverse taxa. *N. alata* and *N. paniculata* belong to different sections of the Nicotianoideae subfamily and have different ploidy levels (Table 5). Despite that phylogenetic distance, *N. paniculata* sets seeds when crossed with *N. alata*, although the hybrid seedlings have poor survival [29]. Their HD-AGPs are only 96% similar, yet, as predicted by the hypothesis, their HV2 sequence and XKPP motifs are essentially identical.

HD-AGPs from *N. obtusifolia* and *N. tomentosiformis* are only 95% identical, but their HV2 domains have not diverged. As predicted by the hypothesis, the two species interbreed readily to produce viable seedlings [31]. The correlation between HV2 divergence and incongruity is readily seen in these two species: They do not hybridize with *N.alata*, *N. paniculata*, or *N. sylvestris* [29,31], and consistent with the hypothesis, the HD-AGP sequences of the two non-hybridizing groups are markedly different in the HV2 region with respect to the number and location of XKPP motifs.

Interspecific barriers in *Nicotiana* are complicated by the prevalence of allopolyploids, which make up about half of all the species in the genus [43]. Although *N. sylvestris* and *N. tomentosiformis* do not hybridize readily, a presumably rare hybridization event followed by polyploidization generated allotetraploid *N. tabacum* about 0.2 million years ago [43]. The presence of HD-AGP homeologs from both ancestral species, *Ns*PRP1 (=TTS1) from *N. sylvestris* and *Nt*PRP1 (=TTS 2) from *N. tomentosiformis*, explains why *N. sylvestris* and *N. tomentosiformis* pollen readily set seed on *N. tabacum* pistils. Allopolyploids tend toward generalist pollination syndromes and usually have short corolla tubes [43], which would further explain their tendency to set seed in a wide variety of heterospecific crosses [49].

### 3.5. Incongruity in Most Solanum Species-Pairs Is also Associated with Variation in XKPP Repeat Motifs

We compared HD-AGP sequences for *Solanum lycopersicum* and *S. pimpinellifolium* and found them to be nearly identical (Figure 6). These two red-fruited species of tomato cross-hybridize easily in both directions with high seed set [33,50].

*S. pennelli* displays unilateral IRBs in crosses with the red-fruited species: It rejects *S. lycopersicum* pollen in an SI-independent manner, but the reciprocal cross yields seed in artificial pollinations [33]. The HD-AGPs of *S. lycopersicum* and *S. pennellii* are strikingly similar, but for a single XKPP indel in the HV2 domain. If a single XKPP can generate incongruity, as appears to be the case in the *P. axillaris axillaris* X *P. integrifolia* cross, why are *S. pennellii* pollen successful in siring seed on *S. lycopersicum* flowers? Rick [51] has noted that seed set in such pollination is low compared to the seed set in conspecific pollination. Furthermore, the two species co-occur in coastal Peru, but natural hybrids between the two have not been found.

We propose that *S. pennellii* pollen tubes do indeed experience incongruence in *S. lycopersicum* pistils because of divergence in a single XKPP motif, resulting in lower pollen tube growth rates than in conspecific pollination. However, because the pistils of *S. lycopersicum* are relatively short, enough of the *S. pennellii* pollen tubes reach the ovary in artificial pollination that some ovules are fertilized before the flower senesces. Lee et al. [30] noted that *Nicotiana* species with long pistils tend to have higher rates of pollen tube growth than species that have short pistils, and they proposed that the length of the pistil may constitute a reproductive barrier in crosses involving long-pistil species X short-pistil species. *S. pennellii* has one of the longest pistils in the *Solanum* clade [30]. The need for HD-AGP congruence in pollen-pistil interactions could also explain conspecific pollen precedence, the phenomenon in which conspecific pollen is more effective in seed siring than is heterospecific pollen when the two are in competition in the same pistil [16]. In nature, pollinator preference combined with conspecific pollen precedence may be adequate for isolating *S. lycopersicum* and *S. pimpinellifolium* from fast-growing *S. pennellii* pollen.

*S. lycopersicoides* is a basal species in the Lycoperison section of the *Solanum* clade. As such, its HD-AGP is divergent from the HD-AGPs of all three of the other *Solanum* species in the multiple alignments in Figure 7. Most of the sequence divergence lies in the HV2 domain, which notably possesses two XKPP indels not seen in the other HD-AGPs in the multiple alignments. As predicted by the hypothesis, this wild tomato does not cross with the other *Solanum* species [52].

### 3.6. There Is Positive Selection on HV1 and HV2, with Purifying Selection on Other HD-AGP Domains

Because all parts of a protein are not necessarily under the same functional and evolutionary constraints, we computed Ka/Ks ratios separately for each of the major domains of HD-AGPs. To examine the hypothesis that HD-AGPs evolve rapidly, with some domains under positive selection and others under evolutionary constraint, we computed the Ka/Ks ratios for each domain using pairwise comparisons of full-length HD-AGPs from 5 divergent members of the Solanaceae. Although the Ka/Ks test for evolutionary change is not highly informative for closely related species (as seen in Table 3), it was very effective in revealing adaptive selection in the hypervariable domains of more distant HD-AGPs (Table 7).

The conserved domains of the proteins are clearly under strong functional constraints. In all pairwise comparisons, the histidine-rich domain and the PAC domain displayed Ka/Ks ratios under 1.00, indicating purifying selection on those domains (Table 7). In contrast, the hypervariable domains exhibited Ka/Ks ratios in excess of 1.0 in these reproductively isolated taxa (Table 7). As adaptively evolving sites, these hypervariable domains are likely candidates for regions of the protein that interact in a species-specific manner with a pollen tube partner.

The *Capsicum* and tomato HD-AGPs were the only sequence-pair in the alignment that did not return Ka/Ks > 1 in the variable domains, even though they exhibit a very divergent HV2 sequence including three XKPP indels. The pepper genome is three times larger than the tomato genome, due to the accumulation of ty3/Gypsy like elements in the euchromatin according to Park et al. [53], and that evolutionary history may have led to an unusually high rate of synonymous mutations which are known to vary among genes and which tend to be high in GC-poor regions [54].

### 3.7. Variation in XKPP Motifs Co-Varies with the Presence of Reproductive Barriers

It may be argued that the link between XKPP variants and interspecific reproductive barriers is simply a proxy for the link between overall phylogenetic divergence and the emergence of reproductive barriers between a pair of species. As HD-AGP sequences diverge overall, according to the argument, so do XKPP motifs, and the resulting mismatch in pollen-pistil interactions then manifests as an interspecific incongruity. However, our statistical tests reveal that XKPP variants are a stronger predictor of reproductive barriers in 15 species-pairs belonging to the Solanaceae than is the overall amino acid similarity between the HD-AGPs of these species.

When considered together, our findings support the hypothesis that variation in the XKPP repeat motif is a significant driver of interspecific barriers to gene flow. They also suggest an important role for these motifs in species-specific interactions between pollen tubes and the pistil. We propose that the HV2 domain is exposed to selective pressure when an ancestral population diverges into two sympatric lineages. With sufficient sequence divergence in the variable domains, mating interactions between the splitting populations fail, generating a pre-zygotic reproductive barrier.

After a speciation event and through the persistence of a species, however, these multi-domain proteins must come under strong purifying selection. Polymorphisms within populations that lead to non-synonymous substitutions are predicted to be deleterious, and, are therefore, likely to be rapidly eliminated from the population. As described in Results 2.11, within-species polymorphism in HD-AGPs is extremely low. These reproductive genes must evolve in spurts, with rapid evolutionary change during speciation events, followed by a period of stasis that could last as long as the species does.

### 3.8. Reinforcement of Sympatric Speciation by Postmating Prezygotic IRBS: A Model

An explosive adaptive radiation that began approximately 49 million years ago has given rise to the 98 extant genera of the Solanaceae, and its approximately 3000 species. Populations diverging in sympatry must acquire breeding barriers rapidly for sustained differentiation of the lineages. The prevalence of the SI X SC rule suggests that co-opting the SI system to block gene flow between the diverging taxons is the fastest means to that end. The mechanism underlying SI-related IRBs is not understood, but it must involve a locus that can mutate rapidly to direct the inhibition of heterospecific pollen of all S-haplotypes, while keeping the infraspecific SI system intact.

It seems unlikely that only SI lineages are routinely protected from maladaptive gene flow and loss of reproductive potential to low fitness hybrids during sympatric speciation. SI-related IRBs, however, are not an option for a diverging lineage that is self-compatible (SC). We propose that incongruity in pollen-pistil interactions caused by HD-AGP divergence is a key mechanism that shelters an SC population from maladaptive hybridization with an incipient sister species. In contrast with SI-related IRBs, reinforcement of speciation by incongruity is easily reversed. Phenotypic plasticity in pistil lengths in a population alone could reverse the barrier.

According to this model of speciation reinforcement, a single mutational event that generates an XKPP indel can alter the functionality of an HD-AGP in pollen-pistil interactions. The presence of contiguous 12-bp repeats in the HV2 domain makes the DNA prone to strand slippage during replication [55], increasing the odds of mutational insertions and deletions of the XKPP motif. Thus, adaptive evolution in the gene can potentially be wrought by a single mutational event yielding a large phenotypic effect. The hypothetical pollen partner of HD-AGPs must co-evolve rapidly as well to maintain congruence in pollen-pistil interactions within the lineage. The transmitting tissue of the pistil is a powerful screen for haplosufficient pollen, and it is not difficult to imagine that cognate pollen genotypes will be swiftly selected through their seed siring success.

During allopatric speciation, or in the absence of speciation, HD-AGPs are under strong purifying selection because knockout phenotypes are quickly weeded out from the gene pool. The model predicts that sympatric sister species isolated through incongruity are likely to exhibit XKPP divergence in the HV2 domain of their HD-AGPs. However, sister species isolated via allopatry, or that underwent polyploid speciation, may possess near-identical HV2 domains, and, therefore, no incongruity-based barriers to reproduction.

## 4. Materials and Methods

### 4.1. Plants

Seeds of *Petunia axillaris axillaris* and *P. integrifolia* were obtained from the Ohio State/USDA Ornamental Plant Germplasm Center. A commercial accession of *P. integrifolia* seeds was purchased from Select Seeds (Union, CT, USA) and commercial accessions of *Petunia axillaris axillaris* and *P. exserta* were purchased from Plant World Seeds (Devon, UK). Plants were grown in a greenhouse on a 16-h photoperiod and supplemental lighting from sodium vapor and metal halide lamps and temperatures ranging from 18–30 °C over the seasons.

### 4.2. Time Course of Pollen Tube Growth Following Conspecific and Heterospecific Pollinations

*P. axillaris* flowers were emasculated 2–3 days prior to anthesis and pollinated by dabbing the stigmatic surface with freshly dehisced anthers until the entire surface was visibly covered with pollen. Pistils (stigma + style) were removed at various times after pollination and immediately fixed in 3:1 ethanol-acetic acid (70% ethanol; glacial acetic acid). Fixed pistils were macerated in 8N KOH overnight, rinsed three times in distilled water, then stained with cleared aniline blue (0.5% aniline blue in 0.1M K_3_PO_4_) to visualize pollen tubes. Each pistil was mounted on a slide in a drop of glycerin before applying the cover slip and viewing with an Olympus BX-40 fluorescence microscope with epifluorescence excitation at 365 nm.

### 4.3. RNA Isolation and DNA Sequencing

Pistils (stigma + style) were removed from emasculated flowers and flash-frozen in liquid nitrogen. Total RNA was extracted from 150 mg fresh weight of pistils with the TōTALLY RNA Total RNA Isolation kit (Ambion, Austin, TX, USA). The mRNA was eluted with nuclease-free water and stored at −80 °C until used.

Total RNA served as a template using the ThermoScript RT-PCR System (Invitrogen, Carlsbad, CA, USA) which relies on an avian reverse transcriptase to extend the Oligo(dT)20 reverse primer. The cDNA was stored at −20 °C. The list of PCR primers is included in Supplementary Materials Table S2.

PCR was conducted with the Platinum Taq DNA Polymerase kit (Invitrogen, Carlsbad, CA, USA) in an Applied Biosystems 2720 thermal cycler. To amplify the entire gene, a gene specific primer (2PhTTS2) along with the Oligo(dT)20 reverse (R) primer were used. The actin-F primer and actin-R primer were used as a positive control for all reactions to ensure successful PCR and to check the cDNA quality. A no-template reaction served as the negative control.

PCR amplicons were purified and cloned separately into pENTR 5′-TOPO TA vector (Invitrogen, Carlsbad, CA, USA) according to the manufacturer’s protocols. The TOPO cloning reactions were transformed into DH5α *Escherichia coli*, and DNA was eluted with nuclease-free water and stored at −20 °C. Plasmids were assayed for gene insert using M13 F and M13 R primers, and plasmid DNA was sent for sequencing at Nevada Genomics (Reno, NV, USA) along with vector primers (M13 F and M13 R) and gene-specific primers (2PhTTS-F and 3PhTTS-R). The sequences were submitted to GenBank (NCBI), and they were assigned the following accession numbers: MK886489 for *PapPRP1*, and MK886488 for *PitPRP1*.

Introns were amplified from genomic DNA using gene-specific primers, and sequenced using nested primers predicted to flank the intron. Intron sequencing was conducted at MCLAB Molecular Cloning Laboratories (San Francisco, CA, USA). Primer sequences are given in Supplementary Materials, Table S2.

### 4.4. Sequence Analysis

The ExPASy program [56] was used to convert the cladogram results from Nevada Genomics (Reno, NV). Sequence alignment, editing, and manipulation were done using Clustal Omega v1.2 and MEGA7 [57]. Nucleotide sequences were confirmed using two different plasmids and four different primers, two gene-specific (2PhTTS F and 3PhTTS R) and two vector primers (M13 F and M13 R), to ensure sequence accuracy. Additional HD-AGP orthologs were obtained from the NCBI database [58] and from Brooks [26]. Twomey et al. [22] and Noyszewski et al. [20] deduced that Z16403.1 (*TTS-1*) has an additional cytosine (C) at position 687 bp from the start codon that creates a frame shift. We deleted C687 to restore the open reading frame, which restored the sequence similarity between *TTS-1* and *TTS-2* [Z16404.1].

### 4.5. Bioinformatics and Statistical Analysis

Pairwise comparisons were conducted using the DnaSP software [59] to calculate the synonymous (Ka) and non-synonymous (Ks) substitution ratio of cDNA sequences [60]. To calculate the Ka/Ks ratio, the Nei-Gojobori method was used, and all positions containing gaps and missing data were eliminated.

We used two-tailed nonparametric tests of association to ask whether the presence of reproductive barriers is best predicted (1) by the presence XKPP variants, or (2) by the overall sequence similarity of HD-AGPs in 25 pairwise comparisons involving 15 solanaceous taxa. We prepared a matrix of pairwise comparisons of HD-AGPs (Table S1, Supplementary Materials) in which the absence/presence of reproductive barriers was scored as 0/1 and comprised the dependent variable. The two independent variables we tested were (1) absence/presence of XKPP variants, recorded in the matrix as 0/1, and (2) percent amino acid identity between the pair of HD-AGPs being compared. Results from Cramer’s V and Spearman’s Rho tests are reported, although Pearson’s R and Kendall’s Tau C returned highly similar values for both association and significance.

### 4.6. Evolutionary Analysis

Supplementary Materials Figure S1 contains all cDNA sequences used for evolutionary analysis. Pairwise comparisons were conducted using the DnaSP software [59] to calculate the non-synonymous (Ka) and synonymous (Ks) substitution ratio of cDNA sequences [60], using the Nei-Gojobori method. Ka/Ks ratios compute the frequency of substitutions alone, and indels in the sequence are eliminated as part of the protocol. Thus, indel-driven evolutionary change is not reflected in the Ka/Ks metric.

Gene trees were constructed using MEGA 7.0 software and MUSCLE algorithm (default settings) with minor manual adjustments. The evolutionary history of HD-AGPs was inferred using the UPGMA method and the Neighbor-Joining method [61], and the bootstrap test was performed for each tree (1000 replicates). The trees are drawn to scale, with branch lengths in the same units as those of the evolutionary distances used to infer the phylogenetic tree. The evolutionary distances were computed using the Tamura-Nei method [62] and are in the units of the number of base substitutions per site. The rate of variation among sites was modeled with a gamma distribution (shape parameter = 2).

## 5. Conclusions

We compared HD-AGP sequences from five species/subspecies of *Petunia*, five species of *Nicotiana*, five species of *Solanum*, and one member of the *Capsicum* genus. In almost all pairwise comparisons, we found that variation in a tetrapeptide repeat motif is associated with reproductive barriers between the species that the sequences come from.

Ka/Ks ratios were not highly informative for detecting evolutionary pressures on HD-AGP domains at the infraspecific level. When HD-AGPs were compared across genera, however, the Ka/Ks metric readily detected positive selection in the two variable domains, and purifying selection on all other domains, in nearly all pairwise comparisons.

These data support the paradigm that speciation is reinforced when breeding between incipient species is blocked by incongruity arising from Darwinian selection for variation in certain HD-AGP domains. These domains are likely involved in species-specific pollen-pistil interactions, which offers clues about the mechanism through which HD-AGPs promote pollen tube growth in the pistil.

## Figures and Tables

**Table 1:** Overview of histidine domain-arabinogalactan proteins (HD-AGPs) from five species and sub-species of wild *Petunia*. All the petunias shaded in blue hybridize readily in the greenhouse to produce large capsules and seed set comparable to those from conspecific matings. The species shaded in grey readily hybridize with each other in the greenhouse to produce large capsules and seed set comparable to those from conspecific matings. The species shown in blue versus grey are reproductively isolated from each other by SI-related IRBs or incongruity, depending on the direction of the cross. All *Petunia HD-AGP*s identified so far contain a single intron at the same location (near the beginning of the PAC domain).

Species	Self-Incompatibilibility (SI) or Self-Compatibility (SC)	Name of HD-AGP	Length in Basepairs (Number of Amino Acids)	Intron Length (bp)
*Petunia axillaris axillaris *	SC/SI	*Paa*PRP1	768 (256)	1102
*Petunia axillaris parodii *	SC	*Pa*pPRP1	768 (256)	595
*Petunia exserta *	SC	*Pe*PRP1	768 (256)	593
*Petunia integrifolia *	SI	*Pit*PRP1	780 (260)	604
*Petunia inflata *	SI	*Pi*PRP1	780 (260)	601

**Table 2:** Comparison of sequence variation in HD-AGPs from hybridizing and non-hybridizing *Petunia* species. Bilateral IRB refers to the failure to set seed in either direction. The underlying mechanisms, in this case, are SI-related IRB when the SI species is the female partner, and incongruity when the SC species is the female partner. Cross-hybridizing *Petunia* species pairs display little to no sequence variation at the protein level. When non-hybridizing species-pairs are compared, sequence variation is seen mainly in the HV2 domain and is strongly biased toward variation in the number of the XKPP tetrapeptides in this domain.

	HD-AGP Sequence Attributes							
Species Pairs Compared	Name of HD-AGPs Compared	Reproductive Barrier	Overall Sequence Similarity: Nucleotide (Amino Acid)	Total # of Variant Residues	Total # of Variant Residues in HV1	Total # of Variant Residues in HV2	Total # of Variant Residues that Lie in XKPP Motifs	XKPP bias (# of Variant Residues in XKPP/Total # of Variant Residues X100)
*P. axillaris axillaris* vs. *P. axillaris parodii*	*Paa*PRP1 vs. *Pap*PRP1	None	99.3% (99.6%)	1	0	0	0	0
*P. axillaris axillaris* vs. *P. exserta*	*Paa*PRP1 vs. *Pe*PRP1	None	99.5% (100%)	0	0	0	0	0
*P. axillaris axillaris* vs. *P. integrifolia*	PaaPRP1 vs. *Pit*PRP1	Bilateral IRB	97.6% (97.3%)	7	0	6	4	57%
*P. axillaris axillaris* vs. *P. inflata*	*Paa*PRP1 vs. *PiP*RP1	Bilateral IRB	97% (96.9%)	8	0	6	4	50%
*P. integrifolia* vs. *P. inflata*	*Pit*PRP1 vs. *Pi*PRP1	None	99.23% (98.9%)	3	0	0	0	0

**Table 3:** Ka/Ks ratios in pairwise comparisons of *Petunia* HD-AGPs. The domains are: Hypervariable I (HV1), Histidine-rich domain (His), Hypervariable II (HV2), and PAC/Ole-1 domain (PAC). The ratio of Ka/Ks yields an undefined value when the rate of synonymous substitutions (Ks) equals zero, but the rate of non-synonymous substitutions (Ka) is greater than zero; this result is shown as Ka minus Ks > 0, in the table. When they are present, IRBs in these species are bilateral. However, the specific type of IRB depends on the direction of the cross: SI-related IRB appears to be operative when the SI species is the female partner; incongruity is the outcome when the SC species is the pistillate partner.

Species Compared	Reproductive Barrier	Sequence 1	Sequence 2	XKPP Indels in HV2	Ka/Ks Ratio			
					HV1	His	HV2	PAC
*P. axillaris axillaris* vs. *P axillaris parodii*	None	*Paa*PRP1	*Pap*PRP1	0	0.0000	0.0000	0.0000	0.6730
*P. axillaris axillaris* vs. *P. exserta*	None	*Paa*PRP1	*Pe*PRP1	0	0.0000	0.0000	0.0000	Ka-Ks > 0
*P. axillaris axillaris* vs. *P. integrifolia*	SI-related IRB + incongruity	*Paa*PRP1	*Pit*PRP1	1	0.0000	0.0000	Ka-Ks > 0	0.2210
*P. axillaris axillaris* vs. *P. inflata*	SI-related IRB + incongruity	*Paa*PRP1	*Pi*PRP1	1	0.0000	0.0000	Ka-Ks > 0	0.9946
*P. axillaris parodii* vs. *P. exserta*	None	*Pap*PRP1	*Pe*PRP1	0	0.0000	0.0000	0.0000	0.0000
*P. axillaris parodii* vs. *P. integrifolia*	SI-related IRB + incongruity	*Pap*PRP1	*Pit*PRP1	1	0.0000	0.0000	Ka-Ks > 0	0.2346
*P. axillaris parodii* vs. *P. inflata*	SI-related IRB + incongruity	*Pap*PRP1	*Pi*PRP1	1	0.0000	0.0000	Ka-Ks > 0	0.4373
*P. exserta* vs. *P. integrifolia*	SI-related IRB + incongruity	*Pe*PRP1	*Pit*PRP1	1	0.0000	0.0000	Ka-Ks > 0	0.3304
*P. exserta* vs. *P. inflata*	SI-related IRB + incongruity	*Pe*PRP1	*Pi*PRP1	1	0.0000	0.0000	Ka-Ks > 0	0.6595
*P. integrifolia* vs. *P. inflata*	None	*Pit*PRP1	*Pi*PRP1	0	0.0000	0.0000	0.0000	0.5931

**Table 4:** Summary of breeding behavior of *Nicotiana* species and attributes of their HD-AGPs. *N. sylvestris* and *N. paniculata* pollen tubes are rejected in *N. alata* pistils due to SI-related IRBs, but *N. paniculata* pistils accept pollen from *N. alata,* despite the very different ploidy and the classification of the two in different sections of *Nicotiana*. *N. alata* pollen set seed on *N. sylvestris* pistils, although the seeds are inviable. *Nicotiana sylvestris* and *N. tomentosiformis* were the ancestral parents of *N. tabacum*, a natural allopolyploid. As a result, *Ns*PRP1 is essentially identical to TTS1 and *Nt*PRP1 to TTS2, the two homologues described from *N. tabacum* by Cheung et al. [28].

Species	Name of HD-AGPs Compared	Section, Haploid Number	SI: Self-Incompatible; SC: Self-Compatible	Breeding Behavior	Length of HD-AGP (# of Amino Acids)	Total # of XKPP Motifs
*Nicotiana alata*	*Na*PRP4	Alatae, n = 9	Mostly SI	Rejects most heterospecific pollen via SI	255	7
*Nicotiana sylvestris*	*Ns*PRP1 *=* TTS1	Sylvestres, n = 12	SC	*N. alata* or *N. paniculata* pollen set seed	257	7
*Nicotiana paniculata*	*Np*PRP1	Paniculatae, n = 12	Mostly SC	*N.alata* or *N. sylvestris* pollen set seed	255	8
*Nicotiana tomentosiformis*	*Nt*PRP1 = TTS2	Tomentosae, n = 12	SC	Hybridizes with *N. obtusifolia*, but IRB with other 3	261	9
*Nicotiana obtusifolia*	*Nob*PRP1	Trigonophyllae N = 12	SC	Hybridizes with *N. tomentosiformis*, but IRB with other 3	263	9

**Table 5:** Association of tetrapeptide repeat motifs with reproductive barriers in five species of *Nicotiana*. Species pairs that hybridize in both directions have similar XKPP repeat motifs in their HD-AGPs. Those that have an IRB in at least one direction have at least one variant XKPP motif (indel or variant motif sequence caused by non-synonymous substitutions).

Species Compared	Seq 1	Seq 2	Reproductive Barrier	Percent Identity: Nucleotide (Amino Acid)	Number of XKPP Variants
*N. alata* vs. *N. sylvestris*	*Na*PRP4	*N*sPRP1	SI-related IRB + incongruity	95 (96)	2
*N. alata* vs. *N. paniculata*	*Na*PRP4	*Np*PRP1	None	94 (95)	1
*N. alata* vs. *N. tomentosiformis*	*Na*PRP4	*Nto*PRP1	SI-related IRB + incongruity	91 (92)	1
*N. alata* vs. *N.obtusifolia*	*Na*PRP4	*Nob*PRP1	SI-related IRB + incongruity	90 (92)	1
*N. sylvestris* vs. *N. paniculata*	*Ns*PRP1	*Np*PRP1	None	94 (93)	0
*N. sylvestris* vs. *N. tomentosiformis*	*Ns*PRP1	NtoPRP1	Bilateral incongruity	89 (88)	1
*N. sylvestris* vs. *N.obtusifolia*	*Ns*PRP1	*Nob*PRP1	Bilateral incongruity	88 (93)	1
*N. paniculata* vs. *N. tomentosiformis*	*Np*PRP1	*Nto*PRP1	Bilateral incongruity	92 (95)	1
*N. paniculata* vs. *N.obtusifolia*	*Np*PRP1	*Nob*PRP1	Bilateral incongruity	94 (94)	1
*N. tomentosiformis* vs. *N.obtusifolia*	*Nto*PRP1	*Nob*PRP1	None	95 (95)	0

**Table 6:** Summary of pairwise comparisons of domains in four species of *Solanum*. Bilateral IRB refers to a failure to set seed in either direction, with details of the underlying mechanism not currently elucidated. A question mark denotes uncertainty or conflicting information about the breeding behavior in the current literature.

Species Compared (SI/SC)	Seq 1	Seq 2	Reproductive Barrier	Percent Identity: Nucleotide (Amino Acid)	XKPP Indels (Number)
*S. lycopersicum* (SC) vs. *S. pimpinellifolium* (SC)	*Sl*PRP1	*Spp*PRP1	None	99.9 (100)	0
*S. lycopersicum* (SC) vs. *S. pennellii* (SI)	*Sl*PRP1	*Sp*PRP1	SI-related IRB + incongruity?	97.1 (97.6)	1
*S. lycopersicum* (SC) vs. *S. lycopersicoides* (SI)	*Sl*PRP4	*Sly*PRP1	SI-related IRB + incongruity	94.4 (94.5)	2
*S. pimpinellifolium* (SC) vs. *S. pennellii* (SI)	*Spp*PRP1	*Sp*PRP1	SI-related IRB + incongruity?	97.1 (97.6)	1
*S. pimpinellifolium* (SC) vs. *S. lycopersicoides* (SI)	*Spp*PRP1	*Sly*PRP1	SI-related IRB + incongruity	94.5 (94.5)	2
*S. pennellii* (SI) vs. *S. lycopersicoides* (SI)	*Sp*PRP1	*Sly*PRP1	Bilateral IRB: presumed SI-related	95.6 (96.4)	2

**Table 7:** Summary of Ka/Ks ratios between pairwise comparisons of domains in HD-AGPs from five genera in the Solanaceae. Positive selection is indicated in bold type. In the one case where the value of Ks equaled zero, and the Ka value was greater than zero, the result is written as “Ka-Ks > 0” to signify that all the substitutions in that domain were non-synonymous. The signature of positive selection is seen in all the pairwise comparison except in the comparisons of *Capsicum annuum* and *S. lycopersicum* HD-AGPs (last row).

8				Ka/Ks Ratio			
Species Compared	Seq 1	Seq 2	Percent Identity: Nucleotide (Amino Acid)	HV1	His	HV2	PAC
*Petunia axillaris* vs. *Nicotiana alata*	*Pa*PRP1	*Na*PRP4	83 (72)	1.5007	0.1556	0.7213	0.3914
*Petunia axillaris* vs. *Capsicum annuum*	*Pa*PRP1	*Ca*PRP1	78 (75)	1.6617	0.1009	1.1805	0.3419
*Petunia axillaris* vs. *Solanum lycopersicum*	*Pa*PRP1	*Sl*PRP1	75 (65)	0.8780	0.0972	1.6127	0.2808
*Petunia axillaris* vs. *Solanum tuberosum*	*Pa*PRP1	*Stu*PRP1	77 (67)	1.6635	0.0972	1.7705	0.2540
*Nicotiana alata* vs. *Capsicum annuum*	*Na*PRP4	*Ca*PRP1	80 (79)	1.9018	0.2060	0.4827	0.2924
*Nicotiana alata* vs. *Solanum lycopersicum*	*Na*PRP4	*Sl*PRP1	77 (69)	1.1220	0.0972	0.1643	0.1971
*Nicotiana alata* vs. *Solanum tuberosum*	*Na*PRP4	*Stu*PRP1	78 (71)	2.2642	0.0972	0.3550	0.2039
*Capsicum annuum* vs. *Solanum tuberosum*	*Ca*PRP1	*Stu*PRP1	79 (71)	Ka-Ks > 0	0.3292	1.1589	0.2091
*Capsicum annuum* vs. *Solanum lycopersicum*	*Ca*PRP1	*Sl*PRP1	73 (64)	0.06875	0.3292	0.7712	0.2716

## Supplementary Materials

The following are available online at https://www.mdpi.com/2223-7747/8/7/211/s1, Figure S1: Multiple Alignment of HD-AGP Nucleotide Sequences, Table S1: Covariance Matrix for 15 Solanaceous HD-AGPs, Table S2: Primer Sequences.
