# Supplementary material for: HD-AGPs as Speciation Genes: Positive Selection on a Proline-Rich Domain in Non-Hybridizing Species of Petunia, Solanum, and Nicotiana
Source: Plants (Basel). 2019 Jul 8;8(7):211. doi: 10.3390/plants8070211 (PMC6681252; doi:10.3390/plants8070211)

Multiple alignment of *HD-AGP* cDNA sequences from four genera of the Solanaceaea

**Order of sequences in this document: *Solanum tuberosum* *HD-AGP* compared to *Capscium annuum* *HD-AGP*; five *Petunia* sequences; five *Nicotiana* sequences; four *Solanum* sequences.**

Solanum atggcaaaggcccttgttctttttcagctttcagttttattacttagctcattcacaatt 60

Capsicum atggcaaaggcccttgttctttttcagctttcagtcttatttcttagctcattcgcagtt 60

*********************************** ***** ************ ** **

Solanum gttagccatgctcaagatgagtggtactcattagacaaaaatgttgaccaccttgcacca 120

Capsicum cttagccacggtga---tcactggtactcattagacaaaaatgttgaccaccttccacca 117

******* * * * * * ********************************* *****

Solanum gctcaagcccctaagccacaccaccaccaccaccaccatccccacccccaccccataagt 180

Capsicum gcccaagcccctaagcctcacaagggccaccacccccccccaaaaaattccccagcccct 177

** ************** *** * ******** ** ** * *** *

Solanum t------------ccccagccccttcaccaatttatt----------------------- 205

Capsicum tcaccaatagatactccaactccaccaccagctaagtctccttctcccccaccagctaag 237

* * *** * ** ***** * * *

Solanum -atccaacaaagccaccaactaaagctccaactaagccaccaactaaagctccatataag 264

Capsicum cccccaactccaccaccagctaagcctccatccccaccaccatcaaagccaccaactaag 297

***** ****** **** ***** * ****** * ** * *** ****

Solanum ccaccaacttatagcccatcaaaaccaccagctaagccaccagttaagccaccaacacca 324

Capsicum ccaccagctaagtccccttctccaccaccagcaaagccaccaactaaaccaccaacaccg 357

****** ** * *** ** ********* ********* *** ***********

Solanum tcaccttattatccttctaggaaacctgtagctgtaagaggacttgtttactgcaaacct 384

Capsicum tcaccttattatccttcaaggaaacctgtcattgtaagaggccttgtttactgcaagcct 417

***************** *********** ********* ************** ***

Solanum tgcaagtttagagggattaatactcttaaccaagctaaaccacttcagggagccaaggtg 444

Capsicum tgcaagtatagagggattgaaactctttaccgagctaaaccacttgagggagccgtggtg 477

******* ********** * ****** *** ************* ******** ****

Solanum aagctagtgtgcaacaacaccaaaaagacattagtagaacaggctgaaacagacaagaat 504

Capsicum aagctagtgtgcaaaaactccaaaaagacactagttgaacagggaaagacagacaagaat 537

************** *** *********** **** ******* * ************

Solanum ggattcttctggatccttcctaaactcttaagctcaggagcctaccacaaatgcaaagtg 564

Capsicum ggatacttctggatcatgccaaaactcttgacctcaggagcctaccacaagtgcaaggtg 597

**** ********** * ** ******** * ****************** ***** ***

Solanum tttttagtatcatcaaacaattcttactgtaatgtcccaacaaattacaatgacggcaaa 624

Capsicum ttcttggtctcatcaaacaactcttactgtaatgtcccaacaaatttcaatggtggaaaa 657

** ** ** *********** ************************* ***** ** ***

Solanum tctggtgcactattgaagtacaccccaccagctccagctactcatctccctattaaacca 684

Capsicum tctggtgctttgttgaaatacacaccaccttccaagccaacacctt------------ca 705

******** * ***** ***** ***** * * ** * * **

Solanum cccacccctaaatatgatttctttactgttggacctttcggatttgaagcttcaaagaaa 744

Capsicum gctatcaccaaatttgatgtcttcaccgttgggccatttggatttgagccctcaaagaag 765

* * * * **** **** **** ** ***** ** ** ******** * ********

Solanum gtgccttgcaagaagtactaattaacttggcaaa-ttagaaattaagataggaagggatg 803

Capsicum gtgccttgcaagaagtaataaattaacttggcaaattagaatttaagactatgg-----g 820

***************** *** * * * * ** ****** ****** *

Solanum atgaatgtttaaggagttaatttaagtgttggagaaagaaacaaattaa----------a 853

Capsicum ataaaagtggaagaaagttaactaagtgttggagaaagaaaaatttgtagtaccaccgtc 880

** ** ** *** * * * ******************* * * *

Solanum gattgtgttcttgttatttccattaataaatgatcatgta-------------------- 893

Capsicum gcttgtgtccttgttatt-ccattaataaatgatgagatcgagtactctaaatgatgaag 939

* ****** ********* *************** * *

Solanum --tgaggaaagaagaatgtagtttaattttcttgttttttcaa----tttcgtttatgta 947

Capsicum aggaaattaaagaatgtactagtttattttcttgttttttttttttcaatttctttctgt 999

* ** * * ** *************** * **

Solanum atgtttaaagcccaaacaagtgaaagggatttataa------------------- 983

Capsicum ctgtttaaagcccaaacaagtcaaaaggatttataaagtttctcttctatgttaa 1054

******************** *** **********


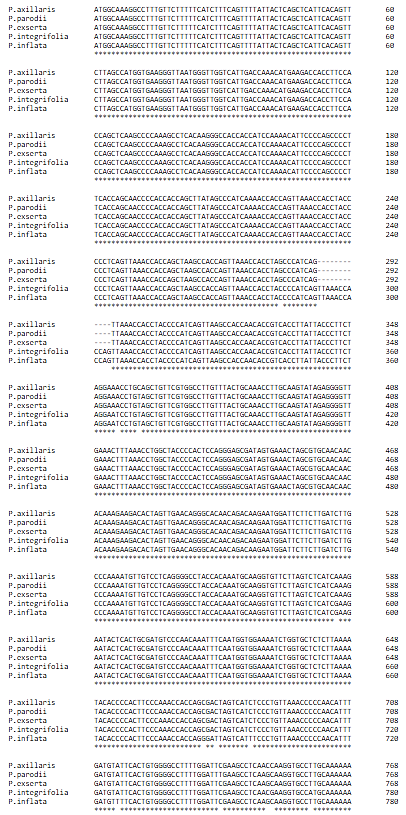


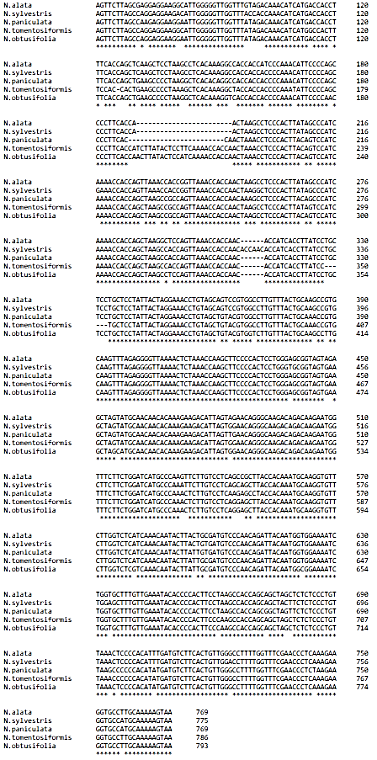

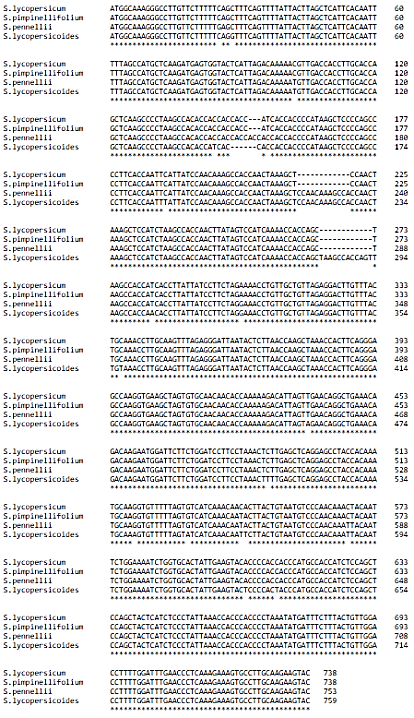

Supplement: Supplementary file 1 [file plants-08-00211-s001.zip › Supplementary Material-final/Supplementary Material.Figure S1.Multiple alignment of HD-AGP nucleotide sequences.docx]
