# Supplementary material for: HD-AGPs as Speciation Genes: Positive Selection on a Proline-Rich Domain in Non-Hybridizing Species of Petunia, Solanum, and Nicotiana
Source: Plants (Basel). 2019 Jul 8;8(7):211. doi: 10.3390/plants8070211 (PMC6681252; doi:10.3390/plants8070211)
Supplement: Supplementary file 1 [file plants-08-00211-s001.zip › Supplementary Material-final/Supplementary Material.Table S1.Covariance matrix for 15 species.docx]

| Species compared | Seq 1 | Seq 2 | **Incongruity** | **XKPP**  **variants** | **Percent similarity** |
| --- | --- | --- | --- | --- | --- |
|  |  |  |  |  | **(%)** |
| *P. axillaris axillaris* vs *P axillaris parodii* | *Pa*PRP1 | *Pa*PRP2 | 0 | 0 | 99.6 |
| *P. axillaris axillaris* vs *P. exserta* | *Pa*PRP1 | *Pe*PRP1 | 0 | 0 | 100 |
| *P. axillaris axillaris* vs  *P. integrifolia* | *Pa*PRP1 | *Pi*PRP1 | + | 1 | 97.3 |
| *P. axillaris axillaris* vs  *P. inflata* | *Pa*PRP1 | *Pif*PRP2 | + | 1 | 96.9 |
| *P. axillaris parodii* vs *P. exserta* | *Pa*PRP2 | *Pe*PRP1 | 0 | 0 | 98.9 |
| *P. axillaris parodii* vs  *P. integrifolia* | *Pa*PRP2 | *Pi*PRP1 | + | 1 | 97.3 |
| *P. axillaris parodii* vs  *P. inflata* | *Pa*PRP2 | *Pif*PRP2 | + | 1 | 96.9 |
| *P. exserta* vs  *P. integrifolia* | *Pe*PRP1 | *Pi*PRP1 | + | 1 | 97.3 |
| *P. exserta* vs  *P. inflata* | *Pe*PRP1 | *Pif*PRP2 | + | 1 | 96.9 |
| *S. lycopersicum vs S. pimpinellifolium* | *Sl*PRP1 | *Spp*PRP1 | 0 | 0 | 100 |
| *S. lycopersicum vs S. pennellii* | *Sl*PRP1 | *Sp*PRP1 | + | 1 | 97.6 |
| *S. lycopersicum vs S. lycopersicoides* | *Sl*PRP4 | *Sly*PRP1 | + | 2 | 93.7 |
| *S. lycopersicum vs S. tuberosum* | *Sl*PRP4 | *Stu*PRP1 | + | 3 | 86.9 |
| *S. pimpinellifolium vs S. pennellii* | *Spp*PRP1 | *Sp*PRP1 | 0* | 1 | 97.6 |
| *S. pimpinellifolium vs S. lycopersicoides* | *Spp*PRP1 | *Sly*PRP1 | + | 2 | 93.7 |
| *S. pennelli vs*  *S. lycopersicoides* | *Sp*PRP1 | *Sly*PRP1 | + | 1 | 96.4 |
| *N. alata vs N. sylvestris* | *Na*PRP4 | *Ns*PRP1 | 0 | 0 | 96 |
| *N. alata vs N. paniculata* | *Na*PRP4 | *Np*PRP1 | 0 | 0 | 95 |
| *N. alata vs N. tomentosiformis* | *Na*PRP4 | *Nto*PRP1 | + | 1 | 92 |
| *N. alata vs N.obtusifolia* | *Na*PRP4 | *Nob*PRP1 | + | 1 | 92 |
| *N. sylvestris vs N. paniculata* | *Ns*PRP1 | *Np*PRP1 | 0 | 0 | 93 |
| *N. sylvestris vs N. tomentosiformis* | *Ns*PRP1 | *Nto*PRP1 | + | 1 | 88 |
| *N. sylvestris vs N.obtusifolia* | *Ns*PRP1 | *Nob*PRP1 | + | 1 | 93 |
| *N. paniculata vs N. tomentosiformis* | *Np*PRP1 | *Nto*PRP1 | + | 1 | 95 |
| *N. paniculata vs N.obtusifolia* | *Np*PRP1 | *Nob*PRP1 | + | 1 | 94 |
| *N. tomentosiformis vs N.obtusifolia* | *Nto*PRP1 | *Nob*PRP1 | 0 | 0 | 95 |

*This species pair is described in the literature as a displaying unilateral IRB (or unilateral incompatibility, UI), implying full compatibility of *S. pennellii* pollen on *S. lycopersicon* pistils. However, other reports describe reduced seed set in such a cross, suggestive of conspecific pollen precedence. For parsimony against the hypothesis, these ambiguous crosses are scored as fully compatible in the reciprocal direction (that is, they are scored as if they uphold the SI X SC rule) in this matrix.
