# Supplementary material for: HD-AGPs as Speciation Genes: Positive Selection on a Proline-Rich Domain in Non-Hybridizing Species of Petunia, Solanum, and Nicotiana
Source: Plants (Basel). 2019 Jul 8;8(7):211. doi: 10.3390/plants8070211 (PMC6681252; doi:10.3390/plants8070211)
Supplement: Supplementary file 1 [file plants-08-00211-s001.zip › Supplementary Material-final/Supplementary Material.Table S2.Primer sequences.docx]

**Primer sequences used to amplify or sequence *Petunia* cDNA and *Petunia* introns**

1. Gene-specific primers

| Type of primer | Name | Primer sequence | Tm  (^o^C) |
| --- | --- | --- | --- |
| Gene-specific, forward | 2PhTTS-F | 5’ CAG TTT TAT TAC TCA GCT CAT TCA CAG TTC 3’ | 63.3 |
| Gene-specific, reverse | 3PhTTS-R | 5' GGC ACC TTS RTT GAG GCT TCG 3' | 65.5 |

B. Intron-flanking nested primers

| Type of primer | Name | Primer sequence | Tm  (^o^C) |
| --- | --- | --- | --- |
| Nested, forward | qRP-F | 5’ GAAACCTGTAGCTGTTCGTGG 3’ | 62.6 |
| Nested, reverse | qPRP-R | 5’ TTGTCTGTTGTGCCCTGTTC 3’ | 60.4 |

C. Control primers corresponding to a *Petunia* actin gene

| Type of primer | Name | Primer sequence | Tm  (^o^C) |
| --- | --- | --- | --- |
| Actin control | Actin-F | 5' ACA GGT ATT GTG TTG GAC TC 3' | 58.4 |
| Actin control | Actin-R | 5' CTG TAC TTT CTC TCT GGT GG 3' | 60.4 |
